# Supplementary material for: Bacterial adaptation to rhizosphere soil is independent of the selective pressure exerted by the herbicide saflufenacil, through the modulation of catalase and glutathione S-transferase
Source: PLoS One. 2023 Nov 14;18(11):e0292967. doi: 10.1371/journal.pone.0292967 (PMC10645333; doi:10.1371/journal.pone.0292967)
Supplement: S1 Appendix — The experimental design indicates the hierarchical order (left to right) of the experiments performed in this article. The microorganisms collected from agricultural soil were submitted to a tolerance test for different concentrations of Heat. The results allowed selecting the strain, identified as Stenotrophomonas sp. CMA 26, which underwent stress indicator tests (growth curve, MDA and H2O2). The response systems degradation and activities of CAT and GST enzymes were evaluated. (DOCX) [file pone.0292967.s001.docx]

S1 Appendix


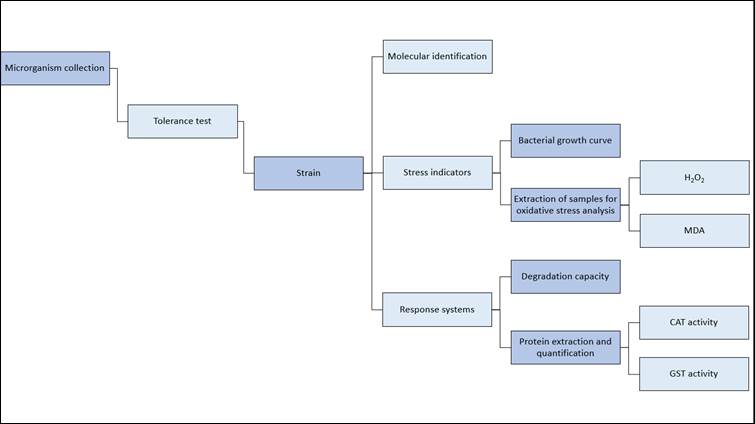


**S1 Appendix: Experimental design.** The experimental design indicates the hierarchical order (left to right) of the experiments performed in this article. The microorganisms collected from agricultural soil were submitted to a tolerance test for different concentrations of Heat. The results allowed selecting the strain, identified as *Stenotrophomonas* sp. CMA 26, which underwent stress indicator tests (growth curve, MDA and H_2_O_2_). The response systems degradation and activities of CAT and GST enzymes were evaluated.
