# Supplementary material for: Bacterial adaptation to rhizosphere soil is independent of the selective pressure exerted by the herbicide saflufenacil, through the modulation of catalase and glutathione S-transferase
Source: PLoS One. 2023 Nov 14;18(11):e0292967. doi: 10.1371/journal.pone.0292967 (PMC10645333; doi:10.1371/journal.pone.0292967)
Supplement: S2 Appendix — Sample collection points per transept, obtained by Global Positioning System (GPS). M7, M8, M9 and M10 represent soybean and corn crop areas. R1, R2, R3, and R4 represent the parallel transepts traced for the collection. (DOCX) [file pone.0292967.s002.docx]

S2 Appendix

**S2 Appendix: Transept localization**

| Area | R1 | R2 | R3 | R4 |
| --- | --- | --- | --- | --- |
| M7 | S 24° 12’ 52.6”  W 053° 54’ 05.2 | S 24° 12’ 54.1”  W 053° 54’ 04.6 | S 24° 12’ 53.9”  W 053° 54’ 02.3 | S 24° 12’ 54.9”  W 053° 54’ 03.9 |
| M8 | S 24° 12’ 46.1”  W 053° 53’ 59.3 | S 24° 12’ 44.2”  W 053° 53’ 59.7 | S 24° 12’ 44.5”  W 053° 54’ 00.8 | S 24° 12’ 42.8”  W 053° 54’ 01.4 |
| M9 | S 24° 17’ 14.3”  W 053° 52’ 30.6 | S 24° 17’ 13.3”  W 053° 52’ 30.5 | S 24° 17’ 12.9”  W 053° 52’ 29.1 | S 24° 17’ 14.1”  W 053° 52’ 28.3 |
| M10 | S 24° 17’ 18.1”  W 053° 53’ 48.4 | S 24° 17’ 18.5”  W 053° 53’ 49.4 | S 24° 17’ 17.6”  W 053° 53’ 49.9 | S 24° 17’ 16.9”  W 053° 53’ 48.1 |

Sample collection points per transept, obtained by Global Positioning System (GPS). M7, M8, M9 and M10 represent soybean and corn crop areas. R1, R2, R3, and R4 represent the parallel transepts traced for the collection.
