# Supplementary material for: Bacterial adaptation to rhizosphere soil is independent of the selective pressure exerted by the herbicide saflufenacil, through the modulation of catalase and glutathione S-transferase
Source: PLoS One. 2023 Nov 14;18(11):e0292967. doi: 10.1371/journal.pone.0292967 (PMC10645333; doi:10.1371/journal.pone.0292967)
Supplement: S3 Appendix — Pesticides used in the collection area. Trademarks of herbicides, fungicides and insecticides and their respective active molecules and chemical structures, modes of action, chemical families, and classification of chemical families according to the Herbicide Resistance Action Committee (HRAC), Fungicide (FRAC) and Insecticide (IRAC) are presented, in addition to the type of culture in which they were used. (DOCX) [file pone.0292967.s003.docx]

S3 Appendix

**S3 Appendix: Chemical characterization of herbicides**

| **Herbicides** | | | | | | | | | |
| --- | --- | --- | --- | --- | --- | --- | --- | --- | --- |
| **Trade mark** | | **Active molecule** | **Chemical structure** | **Action mode** | | **Chemical family** | **Classification HRAC** | | **Culture** |
| Roundup Transorb R | | Gliphosate | 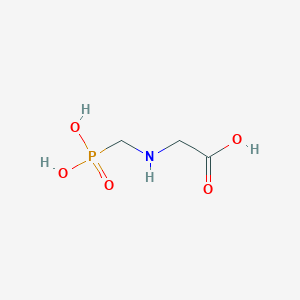 | 5-enolpyruvylshikimate-3-phosphate synthase inhibitor | | Substituted glycine | G | | Soy snd corn |
| Aminol 806 | | 2,4-dichlorophenoxyacetic acid (2,4-D) | 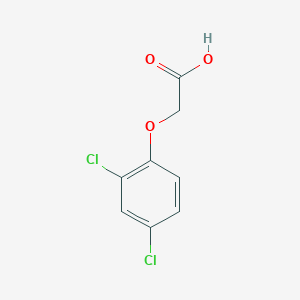 | Auxin mimic | | Phenoxy acids | O | | Soy snd corn |
| Gramoxone 200 | | Paraquat | 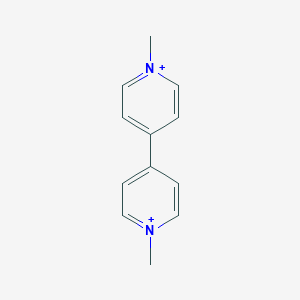 | Photosynthesis inhibitor in photosystem I | | Pyridiniums | D | | Soy |
| Clorimuron Nortox | | Chlorimuron ethyl | 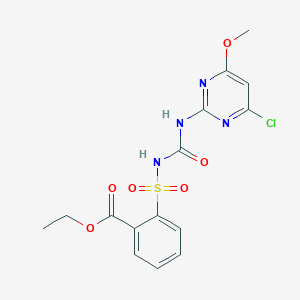 | Acetolactate synthase inhibitor | | Sulfonylureia | B | | Soy |
| Accent | | Nicossulfuron | 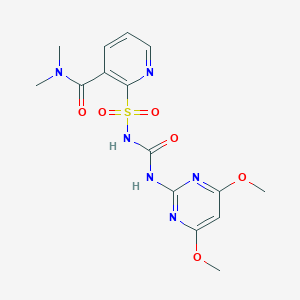 |  |  |  |  |  | Corn |
| Primóleo | | Atrazine | 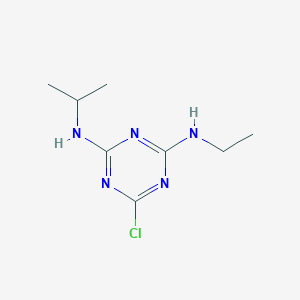 | Photosynthesis inhibitor in photosystem II | | Triazines | C1 | | Corn |
| Callisto | | Mesotrione | 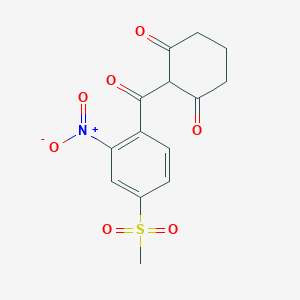 | 4-hydroxyphenyl-pyruvate-dioxygenase inhibitor | | Triketones | F2 | | Corn |
| Soberan | | Tembotrione | 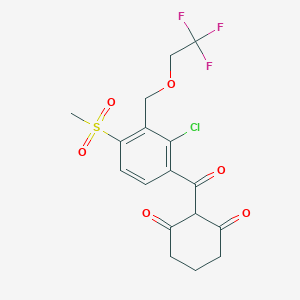 |  |  |  |  |  |  |
| **Fungicides** | | | | | | | | | |
| **Trade mark** | | **Active molecule** | **Chemical structure** | **Action mode** | | **Chemical family** | **Classification FRAC** | | **Culture** |
| Ópera | | Pyraclostrobin | 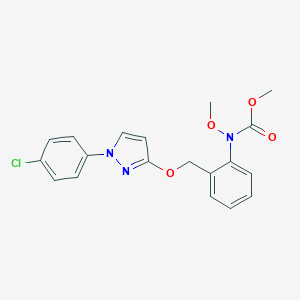 | Cytochrome bc1 inhibitor | | Carbamates | C3 | | Soy snd corn |
|  |  | Epoxiconazole | 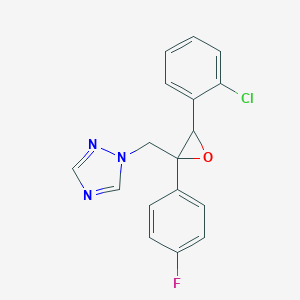 | 14a-demethylase sterol (CYP51) inhibitor | | Triazoles | G1 | |  |
| Priori Xtra | | Azoxystrobin | 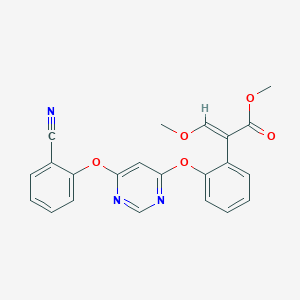 | Cytochrome bc1 inhibitor | | Acrylates | C3 | | Soy |
|  |  | Cyproconazole | 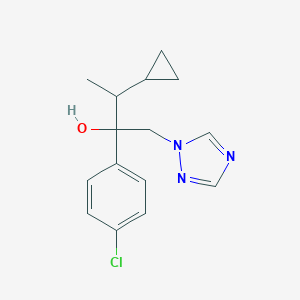 | CYP51 inhibitor | | Triazoles | G1 | |  |
| Tilt | | Propiconazole | 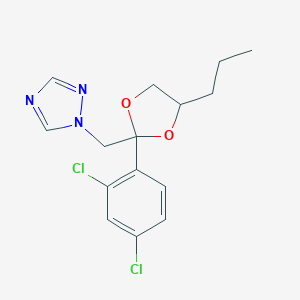 | CYP51 inhibitor | | Triazoles | G1 | | Corn |
| Abacus | | Pyraclostrobin | 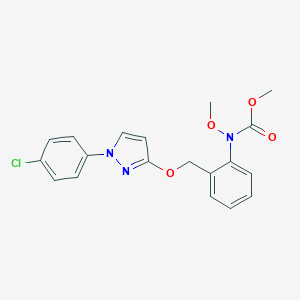 | Cytochrome bc1 inhibitor | | Carbamates | C3 | | Corn |
|  |  | Epoxiconazole | 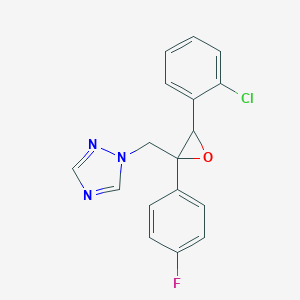 | CYP51 inhibitor | | Triazoles | G1 | |  |
| Aprouch Prima | | Picoxystrobin | 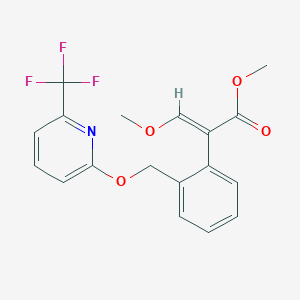 | Cytochrome bc1 inhibitor | | Acrylates | C3 | | Corn |
|  |  | Cyproconazole | 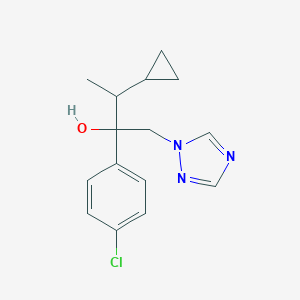 | CYP51 inhibitor | | Triazoles | G1 | |  |
| Sphere Max | | Trifloxystrobin | 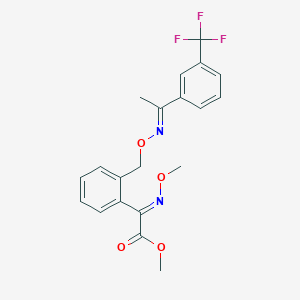 | Cytochrome bc1 inhibitor | | Oxamino acetates | C3 | | Corn |
|  |  | Cyproconazole | 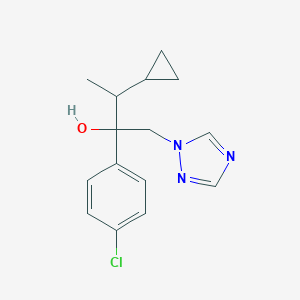 | CYP51 inhibitor | | Triazoles | G1 | |  |
| **Inseticides** | | | | | | | | | |
| **Trade mark** | **Active molecule** | | **Chemical structure** | | **Action mode** | **Chemical family** | **Classification IRAC** | **Culture** | |
| Dimilin | Diflubenzuron | | 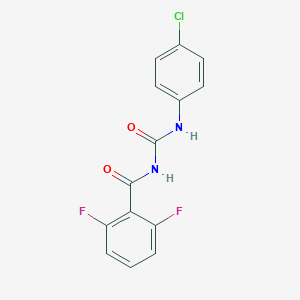 | | Chitin biosynthesis inhibitor | Benzoylureias | 15 | Soy | |
| Thiodan CE | Endosulfan | | 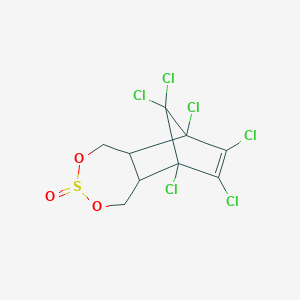 | | GABA controlled chloride channel blockers | Cyclodienochlorines | 2A | Soy | |
| Ampligo | Lambda-cialotrine | | 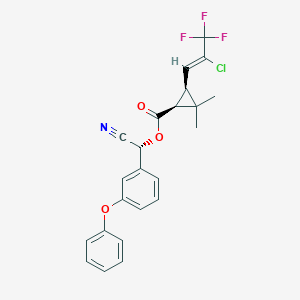 | | Sodium channel modulator | Pyrethrins | 3A | Soy snd corn | |
|  | Chlorantraniliprole | | 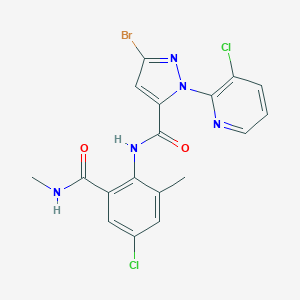 | | Ryanodine receiver modulator | Diamides | 28 |  |  |
| Lannate BR | Metomil | | 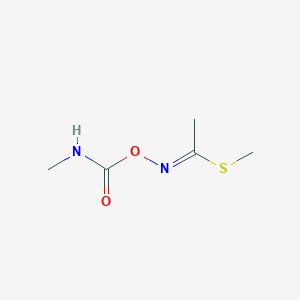 | | Acetylcholinesterase (AChE) inhibitor | Carbamates | 1A | Soy | |
| Engeo pleno S | Thiametoxan | | 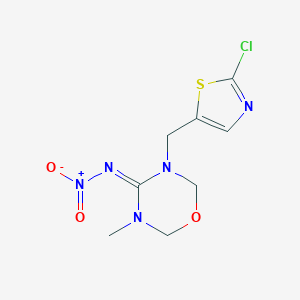 | | Nicotinic acetylcholine receptor (nAChR) modulator | Neonicotinoids | 4A | Corn | |
|  | Lambda-cialotrine | | 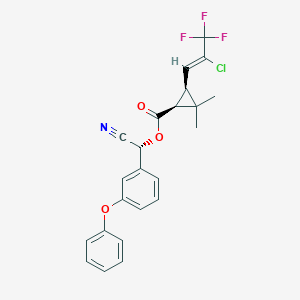 | | Sodium channel modulator | Pyrethrins | 3A |  |  |
| Acefato nortox | Acephate | | 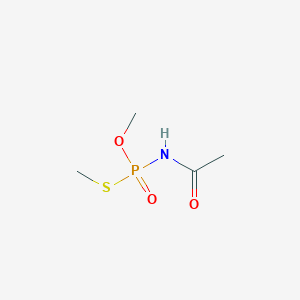 | | AChE inhibitor | Organophosphates | 1B | Corn | |
| Curyom 550 EC | Profenofos | | 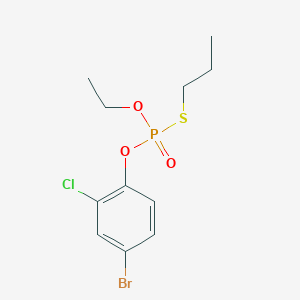 | | AChE inhibitor | Organophosphates | 1B | Corn | |
|  | Lufenuron | | 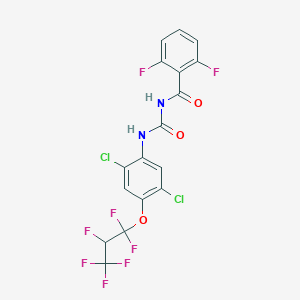 | | Chitin biosynthesis inhibitor | Benzoylureias | 15 |  |  |
| Cropstar | Imidaclopride | | 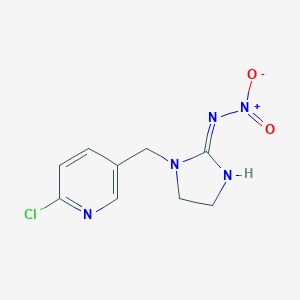 | | nAChR modulator | Neonicotinoids | 4A | Corn | |
|  | Thiodicarb | | 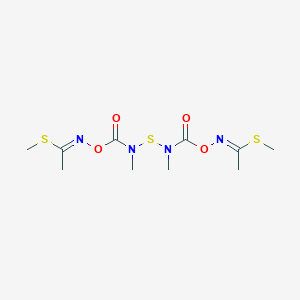 | | AChE inhibitor | Carbamates | 1ª |  |  |
| Cruiser 350 FS | Thiametoxan | | 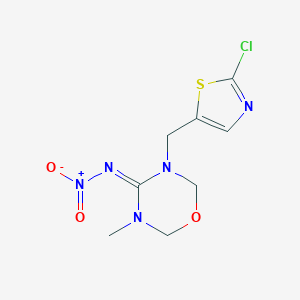 | | nAChR modulator | Neonicotinoids | 4A | Corn | |
| Poncho | Clothianidin | | 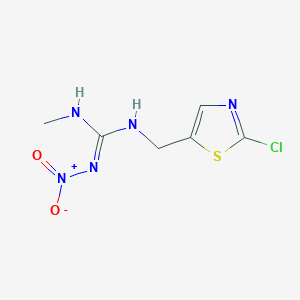 | | nAChR modulator | Neonicotinoids | 4A | Corn | |

Pesticides used in the collection area. Trademarks of herbicides, fungicides and insecticides and their respective active molecules and chemical structures, modes of action, chemical families, and classification of chemical families according to the Herbicide Resistance Action Committee (HRAC), Fungicide (FRAC) and Insecticide (IRAC) are presented, in addition to the type of culture in which they were used.
