# Supplementary material for: Bacterial adaptation to rhizosphere soil is independent of the selective pressure exerted by the herbicide saflufenacil, through the modulation of catalase and glutathione S-transferase
Source: PLoS One. 2023 Nov 14;18(11):e0292967. doi: 10.1371/journal.pone.0292967 (PMC10645333; doi:10.1371/journal.pone.0292967)
Supplement: S4 Appendix — (DOCX) [file pone.0292967.s004.docx]

S4 Appendix

STATISTICAL ANALYSIS FOR GROWTH CURVE

TUKEY TEST TO COMPARE MEANS

Confidence level: 0.95

Dependent variable: zero

Variation Coefficient: 2.775558e-14 %

Independent variable: Trat

Factors Means

0x 0.05 a

10x 0.05 a

1x 0.05 a

50x 0.05 a

TUKEY TEST TO COMPARE MEANS

Confidence level: 0.95

Dependent variable: um

Variation Coefficient: 6.642054 %

Independent variable: Trat

Factors Means

50x 0.162666666666667 a

10x 0.057 b

1x 0.0543333333333333 b

0x 0.0446666666666667 b

TUKEY TEST TO COMPARE MEANS

Confidence level: 0.95

Dependent variable: dois

Variation Coefficient: 9.266254 %

Independent variable: Trat

Factors Means

50x 0.180666666666667 a

1x 0.155 ab

10x 0.139333333333333 b

0x 0.134333333333333 b

TUKEY TEST TO COMPARE MEANS

Confidence level: 0.95

Dependent variable: tres

Variation Coefficient: 11.32057 %

Independent variable: Trat

Factors Means

1x 0.519333333333333 a

0x 0.514 a

10x 0.478333333333333 a

50x 0.284 b

TUKEY TEST TO COMPARE MEANS

Confidence level: 0.95

Dependent variable: quatro

Variation Coefficient: 4.973888 %

Independent variable: Trat

Factors Means

1x 1.007 a

10x 0.969 a

0x 0.902666666666667 a

50x 0.673 b

TUKEY TEST TO COMPARE MEANS

Confidence level: 0.95

Dependent variable: cinco

Variation Coefficient: 4.85553 %

Independent variable: Trat

Factors Means

10x 1.39066666666667 a

0x 1.356 a

1x 1.34266666666667 a

50x 0.9365 b

TUKEY TEST TO COMPARE MEANS

Confidence level: 0.95

Dependent variable: seis

Variation Coefficient: 8.503868 %

Independent variable: Trat

Factors Means

10x 1.79933333333333 a

1x 1.65333333333333 a

0x 1.57733333333333 a

50x 1.559 a

TUKEY TEST TO COMPARE MEANS

Confidence level: 0.95

Dependent variable: sete

Variation Coefficient: 8.295559 %

Independent variable: Trat

Factors Means

10x 2.15766666666667 a

1x 2.048 a

0x 2.014 a

50x 1.95333333333333 a

TUKEY TEST TO COMPARE MEANS

Confidence level: 0.95

Dependent variable: oito

Variation Coefficient: 8.559674 %

Independent variable: Trat

Factors Means

10x 2.332 a

0x 2.288 a

1x 2.259 a

50x 2.172 a

TUKEY TEST TO COMPARE MEANS

Confidence level: 0.95

Dependent variable: nove

Variation Coefficient: 8.464004 %

Independent variable: Trat

Factors Means

10x 2.661 a

1x 2.521 a

50x 2.466 a

0x 2.444 a

TUKEY TEST TO COMPARE MEANS

Confidence level: 0.95

Dependent variable: dez

Variation Coefficient: 11.53567 %

Independent variable: Trat

Factors Means

10x 2.893 a

1x 2.73 a

50x 2.723 a

0x 2.564 a

TUKEY TEST TO COMPARE MEANS

Confidence level: 0.95

Dependent variable: onze

Variation Coefficient: 6.592803 %

Independent variable: Trat

Factors Means

50x 3.046 a

0x 3.014 a

10x 2.993 a

1x 2.848 a

TUKEY TEST TO COMPARE MEANS

Confidence level: 0.95

Dependent variable: doze

Variation Coefficient: 7.216525 %

Independent variable: Trat

Factors Means

10x 3.151 a

0x 3.075 a

50x 3.065 a

1x 2.984 a

TUKEY TEST TO COMPARE MEANS

Confidence level: 0.95

Dependent variable: treze

Variation Coefficient: 7.637474 %

Independent variable: Trat

Factors Means

50x 3.238 a

10x 3.107 a

0x 3.071 a

1x 3.007 a

STATISTICAL ANALYSIS FOR CELL VIABILITY

TUKEY TEST TO COMPARE MEANS

Confidence level: 0.95

Dependent variable: Viabilidade

Variation Coefficient: 16.50856 %

Independent variable: Trat

Factors Means

0 x 4h 6.3e+07 a

0 x 6h 48500000 a

0 x 8h 33500000 a

TUKEY TEST TO COMPARE MEANS

Confidence level: 0.95

Dependent variable: Viabilidade

Variation Coefficient: 17.06872 %

Independent variable: Trat

Factors Means

1x 4h 1.24e+08 a

1x 6h 3.4e+07 b

1x 8h 3.4e+07 b

TUKEY TEST TO COMPARE MEANS

Confidence level: 0.95

Dependent variable: Viabilidade

Variation Coefficient: 15.84707 %

Independent variable: Trat

Factors Means

10x 4h 5.2e+07 a

10x 6h 4.8e+07 a

10x 8h 4.5e+07 a

TUKEY TEST TO COMPARE MEANS

Confidence level: 0.95

Dependent variable: Viabilidade

Variation Coefficient: 9.732194 %

Independent variable: Trat

Factors Means

50x 8h 6.5e+07 a

50x 6h 40500000 b

50x 4h 33500000 b

TUKEY TEST TO COMPARE MEANS

Confidence level: 0.95

Dependent variable: Viabilidade

Variation Coefficient: 17.92539 %

Independent variable: Trat

Factors Means

1x 4h 1.24e+08 a

0x 4h 6.3e+07 b

10x 4h 5.2e+07 b

50x 4h 33500000 b

TUKEY TEST TO COMPARE MEANS

Confidence level: 0.95

Dependent variable: Viabilidade

Variation Coefficient: 11.87005 %

Independent variable: Trat

Factors Means

0x 6h 48500000 a

10x 6h 4.8e+07 a

50x 6h 40500000 a

1x 6h 3.4e+07 a

| TUKEY TEST TO COMPARE MEANS    Confidence level: 0.95  Dependent variable: Viabilidade  Variation Coefficient: 10.47948 %    Independent variable: Trat  Factors Means  50x 8h 6.5e+07 a  10x 8h 4.5e+07 b  1x 8h 3.4e+07 b  0x 8h 33500000 b |
| --- |

STATISTICAL ANALYSIS FOR H_2_O_2_

TUKEY TEST TO COMPARE MEANS

Confidence level: 0.95

Dependent variable: H2O2

Variation Coefficient: 39.97849 %

Independent variable: Trat

Factors Means

0 x 8h 0.65493 a

0 x 4h 0.465728 a

0 x 6h 0.118216 a

TUKEY TEST TO COMPARE MEANS

Confidence level: 0.95

Dependent variable: H2O2

Variation Coefficient: 22.70014 %

Independent variable: Trat

Factors Means

1x 8h 0.9993745 a

1x 4h 0.742254 ab

1x 6h 0.203756 b

TUKEY TEST TO COMPARE MEANS

Confidence level: 0.95

Dependent variable: H2O2

Variation Coefficient: 24.03402 %

Independent variable: Trat

Factors Means

10x 6h 1.154617 a

10x 4h 0.7519565 ab

10x 8h 0.1940535 b

TUKEY TEST TO COMPARE MEANS

Confidence level: 0.95

Dependent variable: H2O2

Variation Coefficient: 25.59115 %

Independent variable: Trat

Factors Means

50x 4h 1.0575905 a

50x 6h 0.829578 a

50x 8h 0.305634 a

TUKEY TEST TO COMPARE MEANS

Confidence level: 0.95

Dependent variable: H2O2

Variation Coefficient: 19.74811 %

Independent variable: Trat

Factors Means

50x 4h 1.0575905 a

10x 4h 0.7519565 a

1x 4h 0.742254 a

0x 4h 0.465728 a

TUKEY TEST TO COMPARE MEANS

Confidence level: 0.95

Dependent variable: H2O2

Variation Coefficient: 34.84237 %

Independent variable: Trat

Factors Means

10x 6h 1.154617 a

50x 6h 0.829578 ab

1x 6h 0.203756 b

0x 6h 0.118216 b

TUKEY TEST TO COMPARE MEANS

Confidence level: 0.95

Dependent variable: H2O2

Variation Coefficient: 27.31084 %

Independent variable: Trat

Factors Means

1x 8h 0.9993745 a

0x 8h 0.65493 ab

50x 8h 0.305634 b

10x 8h 0.1940535 b

STATISTICAL ANALYSIS FOR MDA

TUKEY TEST TO COMPARE MEANS

Confidence level: 0.95

Dependent variable: MDA

Variation Coefficient: 22.47043 %

Independent variable: Trat

Factors Means

0 x 6h 2.98375 a

0 x 4h 2.44125 a

0 x 8h 1.97625 a

TUKEY TEST TO COMPARE MEANS

Confidence level: 0.95

Dependent variable: MDA

Variation Coefficient: 13.86026 %

Independent variable: Trat

Factors Means

1x 4h 4.37875 a

1x 8h 3.68125 ab

1x 6h 2.324925 b

TUKEY TEST TO COMPARE MEANS

Confidence level: 0.95

Dependent variable: MDA

Variation Coefficient: 20.43401 %

Independent variable: Trat

Factors Means

10x 8h 4.84375 a

10x 4h 4.7275 a

10x 6h 1.97625 a

TUKEY TEST TO COMPARE MEANS

Confidence level: 0.95

Dependent variable: MDA

Variation Coefficient: 5.8875 %

Independent variable: Trat

Factors Means

50x 4h 5.69625 a

50x 8h 3.99125 b

50x 6h 3.41 b

TUKEY TEST TO COMPARE MEANS

Confidence level: 0.95

Dependent variable: MDA

Variation Coefficient: 14.31163 %

Independent variable: Trat

Factors Means

50x 4h 5.69625 a

10x 4h 4.7275 ab

1x 4h 4.37875 ab

0x 4h 2.44125 b

TUKEY TEST TO COMPARE MEANS

Confidence level: 0.95

Dependent variable: MDA

Variation Coefficient: 11.13035 %

Independent variable: Trat

Factors Means

50x 6h 3.41 a

0x 6h 2.98375 ab

1x 6h 2.324925 ab

10x 6h 1.97625 b

TUKEY TEST TO COMPARE MEANS

Confidence level: 0.95

Dependent variable: MDA

Variation Coefficient: 18.46276 %

Independent variable: Trat

Factors Means

10x 8h 4.84375 a

50x 8h 3.99125 ab

1x 8h 3.68125 ab

0x 8h 1.97625 b

STATISTICAL ANALYSIS FOR CAT

TUKEY TEST TO COMPARE MEANS

Confidence level: 0.95

Dependent variable: CAT

Variation Coefficient: 1.549039 %

Independent variable: Trat

Factors Means

0 x 8h 518.8492601 a

0 x 6h 219.783655 b

0 x 4h 104.8382558 c

TUKEY TEST TO COMPARE MEANS

Confidence level: 0.95

Dependent variable: CAT

Variation Coefficient: 12.32395 %

Independent variable: Trat

Factors Means

1x 8h 468.3801545 a

1x 6h 147.00392965 b

1x 4h 87.60054817 b

TUKEY TEST TO COMPARE MEANS

Confidence level: 0.95

Dependent variable: CAT

Variation Coefficient: 3.69828 %

Independent variable: Trat

Factors Means

10x 8h 571.3895268 a

10x 4h 125.70863205 b

10x 6h 114.03763375 b

TUKEY TEST TO COMPARE MEANS

Confidence level: 0.95

Dependent variable: CAT

Variation Coefficient: 4.894465 %

Independent variable: Trat

Factors Means

50x 8h 841.1173024 a

50x 4h 144.65907105 b

50x 6h 111.9450775 b

TUKEY TEST TO COMPARE MEANS

Confidence level: 0.95

Dependent variable: CAT

Variation Coefficient: 6.550276 %

Independent variable: Trat

Factors Means

50x 4h 144.65907105 a

10x 4h 125.70863205 ab

0x 4h 104.8382558 bc

1x 4h 87.60054817 c

TUKEY TEST TO COMPARE MEANS

Confidence level: 0.95

Dependent variable: CAT

Variation Coefficient: 5.81778 %

Independent variable: Trat

Factors Means

0x 6h 219.783655 a

1x 6h 147.00392965 b

10x 6h 114.03763375 b

50x 6h 111.9450775 b

TUKEY TEST TO COMPARE MEANS

Confidence level: 0.95

Dependent variable: CAT

Variation Coefficient: 4.783244 %

Independent variable: Trat

Factors Means

50x 8h 841.1173024 a

10x 8h 571.3895268 b

0x 8h 518.8492601 b

1x 8h 468.3801545 b

STATISTICAL ANALYSIS FOR GST

TUKEY TEST TO COMPARE MEANS

Confidence level: 0.95

Dependent variable: GST

Variation Coefficient: 17.64066 %

Independent variable: Trat

Factors Means

0 x 8h 0.0126435075 a

0 x 4h 0.0107039575 a

0 x 6h 0.006463459 a

TUKEY TEST TO COMPARE MEANS

Confidence level: 0.95

Dependent variable: GST

Variation Coefficient: 11.21956 %

Independent variable: Trat

Factors Means

1x 8h 0.0146135515 a

1x 6h 0.0090090635 b

1x 4h 0.005945728 b

TUKEY TEST TO COMPARE MEANS

Confidence level: 0.95

Dependent variable: GST

Variation Coefficient: 9.29996 %

Independent variable: Trat

Factors Means

10x 8h 0.0181326695 a

10x 4h 0.010557746 b

10x 6h 0.005127837 c

TUKEY TEST TO COMPARE MEANS

Confidence level: 0.95

Dependent variable: GST

Variation Coefficient: 16.79654 %

Independent variable: Trat

Factors Means

50x 8h 0.0143823105 a

50x 6h 0.0076745785 b

50x 4h 0.005229246 b

TUKEY TEST TO COMPARE MEANS

Confidence level: 0.95

Dependent variable: GST

Variation Coefficient: 15.65143 %

Independent variable: Trat

Factors Means

0x 4h 0.0107039575 a

10x 4h 0.010557746 a

1x 4h 0.005945728 ab

50x 4h 0.005229246 b

TUKEY TEST TO COMPARE MEANS

Confidence level: 0.95

Dependent variable: GST

Variation Coefficient: 10.48703 %

Independent variable: Trat

Factors Means

1x 6h 0.0090090635 a

50x 6h 0.0076745785 ab

0x 6h 0.006463459 ab

10x 6h 0.005127837 b

TUKEY TEST TO COMPARE MEANS

Confidence level: 0.95

Dependent variable: GST

Variation Coefficient: 12.76078 %

Independent variable: Trat

Factors Means

10x 8h 0.0181326695 a

1x 8h 0.0146135515 a

50x 8h 0.0143823105 a

0x 8h 0.0126435075 a

PCA ANALYSIS GROUPING STATISTICS

EARLY LOG

***VECTORS

PC1 PC2 r2 Pr(>r)

H2O2 0.98541 -0.17023 0.8793 0.003 **

MDA 0.99826 0.05901 0.8730 0.008 **

CAT 0.40599 0.91388 0.8819 0.007 **

GST -0.58581 0.81045 0.7475 0.046 *

---

Signif. codes: 0 ‘***’ 0.001 ‘**’ 0.01 ‘*’ 0.05 ‘.’ 0.1 ‘ ’ 1

Permutation: free

Number of permutations: 999

EARLY-MID LOG

***VECTORS

PC1 PC2 r2 Pr(>r)

H2O2 0.93570 0.35280 0.9851 0.001 ***

MDA -0.35344 0.93546 0.6867 0.073 .

CAT -0.83492 -0.55038 0.9401 0.002 **

GST -0.57401 0.81885 0.6655 0.059 .

---

Signif. codes: 0 ‘***’ 0.001 ‘**’ 0.01 ‘*’ 0.05 ‘.’ 0.1 ‘ ’ 1

Permutation: free

Number of permutations: 999

MID LOG

***VECTORS

PC1 PC2 r2 Pr(>r)

H2O2 -0.95772 0.28771 0.8175 0.019 *

MDA 0.91411 0.40546 0.6871 0.023 *

CAT 0.54622 -0.83764 0.8905 0.004 **

GST 0.51930 0.85459 0.8354 0.006 **

---

Signif. codes: 0 ‘***’ 0.001 ‘**’ 0.01 ‘*’ 0.05 ‘.’ 0.1 ‘ ’ 1

Permutation: free

Number of permutations: 999
