# Supplementary material for: Bacterial adaptation to rhizosphere soil is independent of the selective pressure exerted by the herbicide saflufenacil, through the modulation of catalase and glutathione S-transferase
Source: PLoS One. 2023 Nov 14;18(11):e0292967. doi: 10.1371/journal.pone.0292967 (PMC10645333; doi:10.1371/journal.pone.0292967)
Supplement: S5 Appendix — Cell viability (in CFU) of Stenotrophomonas sp. CMA26 in control (C) and treatments containing 1x, 10x, and 50x the concentration equivalent to that used in the field of herbicide Heat (1x, 10x, and 50x), in the early, early-mid and mid phases of the log. Uppercase letters statistically compare different treatments from the same growth phase; lowercase letters statistically compare the same treatments at different growth stages. The bars represent the standard errors in the means. Tukey’s test (p < 0.05). (DOCX) [file pone.0292967.s005.docx]

S5 Appendix


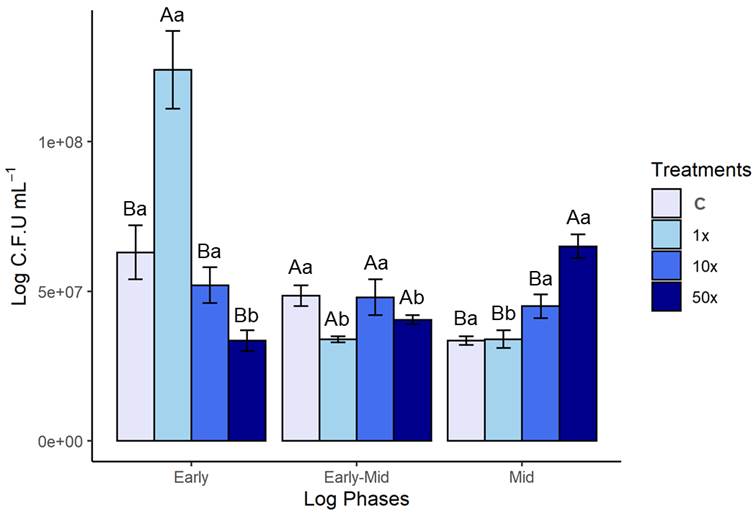


**S5 Appendix: Cell viability.** Cell viability (in CFU) of *Stenotrophomonas* sp. CMA26 in control (C) and treatments containing 1x, 10x, and 50x the concentration equivalent to that used in the field of herbicide Heat (1x, 10x, and 50x), in the early, early-mid and mid phases of the log. Uppercase letters statistically compare different treatments from the same growth phase; lowercase letters statistically compare the same treatments at different growth stages. The bars represent the standard errors in the means. Tukey's test (p < 0.05).
