# Supplementary material for: Bacterial adaptation to rhizosphere soil is independent of the selective pressure exerted by the herbicide saflufenacil, through the modulation of catalase and glutathione S-transferase
Source: PLoS One. 2023 Nov 14;18(11):e0292967. doi: 10.1371/journal.pone.0292967 (PMC10645333; doi:10.1371/journal.pone.0292967)
Supplement: Attachment — Submitted filename: Response to Reviewers.docx [file pone.0292967.s006.docx]

Response to Reviewers

Dear Dr.

Eugenio Llorens

Academic Editor

PLOS ONE

Thank you to the editor and reviewers for evaluating and considering improvements to the submitted manuscript. Please find attached our revised research article entitled “Bacterial Adaptation to Rhizosphere Soil is Independent of the Selective Pressure Exerted by the Herbicide Saflufenacil, through the Modulation of Catalase and Glutathione S-transferase.”

We are submitting a letter that responds to each point raised by the reviewers, the marked-up copy of the revised manuscript with track changes made to the original version, and an unmarked version of the revised paper without tracked changes.

Sincerely, the authors.

Journal requirements:

1. Please ensure that your manuscript meets PLOS ONE's style requirements, including those for file naming. The PLOS ONE style templates can be found at

<https://journals.plos.org/plosone/s/file?id=wjVg/PLOSOne_formatting_sample_main_body.pdf> and

<https://journals.plos.org/plosone/s/file?id=ba62/PLOSOne_formatting_sample_title_authors_affiliations.pdf>

Authors' response: Modifications were made to the manuscript to comply with the style requirements of the journal, as specified in the provide manuals.

2. In your Methods section, please provide additional information regarding the permits you obtained for the work. Please ensure you have included the full name of the authority that approved the field site access and, if no permits were required, a brief statement explaining why.

Authors' response: No permits were required to carry out this work. However, we have added information about the collection of agricultural soil, indicating the person responsible, and have included her as the author. Revised Text: Lines 220–222 of the Revised Manuscript with Track Changes.

3. We note that the grant information you provided in the ‘Funding Information’ and ‘Financial Disclosure’ sections do not match.

When you resubmit, please ensure that you provide the correct grant numbers for the awards you received for your study in the ‘Funding Information’ section.

Authors' response: There was no grant number. The development agency granted a scholarship to the author, Caroline Rosa Silva. Changes have been made to the “Funding Information” and “Financial Disclosure” sections to ensure that the correct information is provided. In the manuscript, the “Funding Information” section has also been added to explain the scholarship grant. Revised Text: Lines 991–993 of the Revised Manuscript with Track Changes.

4. We note that you have included the phrase “data not shown” in your manuscript. Unfortunately, this does not meet our data sharing requirements. PLOS does not permit references to inaccessible data. We require that authors provide all relevant data within the paper, Supporting Information files, or in an acceptable, public repository. Please add a citation to support this phrase or upload the data that corresponds with these findings to a stable repository (such as Figshare or Dryad) and provide and URLs, DOIs, or accession numbers that may be used to access these data. Or, if the data are not a core part of the research being presented in your study, we ask that you remove the phrase that refers to these data.

Authors' response: The phrase has been removed. Revied Text: Line 470 of the Revised Manuscript with Track Changes. We believe that presenting a negative result for herbicide degradation in the manuscript would be of little relevance.

5. Please include captions for your Supporting Information files at the end of your manuscript, and update any in-text citations to match accordingly. Please see our Supporting Information guidelines for more information: <http://journals.plos.org/plosone/s/supporting-information>.

Authors' response: A "Supporting Information" section has been added to the manuscript. In this section, captions were included for the respective files. Revised Text: Lines 1498–1522 of the Revised Manuscript with Track Changes.

Reviewer #1:

Reviewer #1: This study mainly evaluated the tolerance of bacteria Stenotrophomonas sp. CMA26 on herbicide Heat. The authors found that Bacteria CMA26 adaptation is independent of the selective pressure exerted by the herbicide saflufenacil, through the modulation of Catalase and Glutathione S-transferase. The results have some significances for understanding the effects of pesticides on soil microorganisms.

Authors' response: Thank you very much for reviewing the manuscript. We strive to address all the concerns raised by the reviewer.

1. The language needs to be further improved. The article doesn't read smoothly.

Authors' response: The manuscript was revised using the "WORDVICE AI" platform to improve the readability of the text.

2. Abstract Ln32-34, the sentence should be deleted from the abstract. Abstract is not discussion.

Authors' response: The sentence has been deleted, and the abstract has been rewritten. Revised Text: Lines 34–58 of the Revised Manuscript with Track Changes.

3. Ln 58, what is Heat? Is it a pesticide commercial name? If yes, give a standard name.

Authors' response: Yes, Heat is a commercial brand of herbicide, in Brazil that contains saflufenacil as its active ingredient. We have made modifications to the manuscript in order to clarify the definition in the introduction (Lines 99–100 of the Revised Manuscript with Track Changes) and throughout the entire text.

4. Ln 165, where are the section 2.2 and 2.1?

Authors' response: We excluded this sentence from the manuscript because the format does not have numbered sections. Revised Text: Lines 217, 224, 226, 253, 262, 269, and 336 of the Revised Manuscript with Track Changes.

5. Conclusion, please give a clear conclusion. I don't know what the purpose of this work according to the article.

Authors' response: Thank you very much for your review. The conclusions were rewritten to address the objectives of the study, which were to assess the presence of a response system to tolerate Heat herbicide *in vitro*, but not *in situ*, and to examine the implications of this response system on microbial ecology and soil contamination. Revised Text: Lines 917–944 of the Revised Manuscript with Track Changes.

Reviewer #2:

Reviewer #2: Dear Editor,

The current submission deals with the tolerance mechanism of soil-isolated bacteria to HEAT herbicide. Generally, the language of the MS needs to be revised; most of the sentence structures require careful revision. The long title is fine and informative; the short one may be deleted.

Authors' response: Thank you very much for reviewing the manuscript. We strive to address all the concerns raised by the reviewer. The manuscript was revised by the "WORDVICE AI" platform to improve the language structure. The short title has been deleted. Revised Text: Line 7 of the Revised Manuscript with Track Changes.

Abstract:
Lines 27–29: Some sentences are repeated.

Line 42: What does the author mean by ".. originated them..."?

Authors' response: We excluded repeated sentences. The abstract has been rewritten. We would like to clarify that this response system, which is capable of tolerating heat regardless of previous selection for this herbicide, may have evolved in response to reactive oxygen species, regardless of the substance causing the oxidative stress. Revised Text: Lines 52–54 of the Revised Manuscript with Track Changes.

Introduction:
It is OK. However, details on different mechanisms of bacterial tolerance to herbicides should be provided, and references should be updated.

Authors' response: Details on the various mechanisms of bacterial tolerance have been included. Revised Text: Lines 158–159, 159–160, 162, and 163 of the Revised Manuscript with Track Changes. We have also updated the references in the Introduction. Revised Text: Lines 96, 121-122, 131-132, 142, 158–161 of the Revised Manuscript with Track Changes.

Material and Methods:

Suitable methods were applied, but other methods (e.g., gene expression) were needed to uncover the exact mechanism for HEAT herbicide tolerance.

Authors' response: Thank you very much for the suggestion. Methods that include gene expression analysis will undoubtedly enhance data on response systems. However, the intention of this work was to initiate the characterization of a response system by describing the performance of two enzymes, CAT and GST. Our future work will include gene expression analysis to further enhance the robustness of the response system.

Discussion:
It looks like results; the author should really discuss their findings.

Authors' response: The discussion has been expanded. Revised Text: Lines 652–681, 756–757, 760–767, 779–783, 787-790, 853–876, 877–883 of the Revised Manuscript with Track Changes. Fig. 7, which was previously located in the "Discussion" section, has been moved to the "Results" section. Revised Text: Lines 539–542 of the Revised Manuscript with Track Changes.

Conclusion:
OK

Authors' response: Thank you very much for your review.

References:
needs an update with recent references.

Authors' response: The references have been updated. The new references included are in lines 1025–1027, 1031–1033, 1062–1065, 1066–1068, 1140–1142, 1152–1155, 1176–1178, 1190–1192, 1201–1203, 1212–1214, 1215–1219 of the Revised Manuscript with Track Changes.
